# Personalized diagnosis of radiation pneumonitis in breast cancer patients based on radiomics

**DOI:** 10.3389/fonc.2025.1609421

**Published:** 2025-07-22

**Authors:** Xiaobo Wen, Yutao Zhao, Wen Dong, Congbo Yang, Jinzhi Li, Li Sun, Yutao Xiu, Chang’e Gao, Ming Zhang

**Affiliations:** ^1^ School of Pharmacy, Qingdao University, Qingdao, China; ^2^ Department of Radiotherapy, Yunnan Cancer Hospital, the Third Affiliated Hospital of Kunming Medical University, Kunming, Yunnan, China; ^3^ Cancer Institute of The Affiliated Hospital of Qingdao University and Qingdao Cancer Institute, Qingdao, China; ^4^ Department of Medical Oncology, The First Affiliated Hospital of Kunming Medical University, Kunming, China

**Keywords:** breast cancer, radiomics, radiation pneumonitis, machine learning, artificial intelligence

## Abstract

**Objective:**

This study aimed to identify CT‐based radiomic alterations associated with radiation pneumonitis (RP) and to evaluate the feasibility of machine learning classifiers for personalized RP diagnosis in breast cancer patients using these radiomic signatures.

**Methods:**

A total of 146 planning CT scans (pre- and post-radiotherapy) from 73 breast cancer patients with confirmed RP were retrospectively analyzed. The entire lung was delineated as the region of interest (ROI), and 1,834 radiomic features were extracted using PyRadiomics. Feature selection was performed sequentially using the Mann–Whitney U-test (p < 0.05), Spearman’s rank correlation (|ρ| < 0.9), and least absolute shrinkage and selection operator (LASSO). Eight classifiers [logistic regression (LR), support vector machine (SVM), K-nearest neighbor (KNN), random forest (RF), Extra Tree (ET), XGBoost, LightGBM, and multilayer perceptron (MLP)] were trained and evaluated using accuracy, area under the receiver operating characteristic curve (AUC) with 95% confidence intervals, sensitivity, and specificity.

**Results:**

In the independent test cohort, LR achieved the highest performance [accuracy 0.897, AUC 0.929 (95% CI, 0.838–1.000), sensitivity 0.786, and specificity 1.000]. LightGBM and MLP also exhibited robust discrimination with AUC values of 0.855 (95% CI, 0.719–0.990) and 0.848 (95% CI, 0.705–0.991), respectively. Five texture-oriented and four first-order features were retained, underscoring the importance of texture-focused extractors [wavelet and local binary pattern (LBP)].

**Conclusion:**

CT-derived radiomic signatures combined with machine learning classifiers enable the accurate detection of RP in breast cancer patients. Texture-oriented feature selection enhances model discrimination, providing potential for the personalized diagnosis of RP in breast cancer patients and adaptive treatment planning.

## 1 Introduction

The 2020 Global Cancer Statistics found 2.26 million new cases of breast cancer, making it the most common cancer globally, surpassing lung cancer (1). Radiotherapy has become a critical component in treating breast cancer using high-energy rays to prevent the growth or kill cancer cells, leading to reduced recurrence rate and improved quality of life for patients (2–9). However, patients may experience different side effects during the radiation treatment due to the tumor’s location and anatomical structures. Radiation pneumonitis (RP), a prevalent side effect after radiation therapy for breast cancer, impacts subsequent radiation dosages and treatment strategies and significantly influences patients’ quality of life and post-treatment recovery. Therefore, the accurate diagnosis and prediction of RP occurrence can help clinical oncologists adjust treatment plans rapidly, thereby improving patients’ prognosis and quality of life.

Radiomics is a cutting-edge technology that converts region of interest (ROI) image data into high-resolution feature space data that can be easily processed through advanced data characterization algorithms (10). Through radiomics, valuable information can be extracted from image data in an efficient and automated manner, allowing for accurate diagnoses and prognoses. Therefore, radiomics is promising to provide an approach to address the diagnosis and prognosis of RP.

Currently, some studies have attempted to use radiomics to predict the occurrence and grading of RP (11–14). However, a majority of them have focused on the occurrence of RP after radiotherapy for lung cancer treatment, while fewer studies have been conducted on that after radiotherapy for breast cancer. RP after radiotherapy for lung cancer mainly appears in the lobes of the lung where the primary lesion is present. However, radiation-induced RP for breast cancer is mainly in the first to second intercostal space. This difference in location can impact the extracted radiomic features to some extent, affecting the results obtained from radiomics analysis. Therefore, the present study aimed to investigate radiomic feature changes on CT images before and after radiotherapy in breast cancer patients with RP and to assess the potential of these features for diagnosing RP, thereby facilitating improved prognosis and personalized treatment strategies for breast cancer patients.

## 2 Materials and methods

### 2.1 Patient population

This study collected a total of 146 samples, consisting of 73 breast cancer patients’ CT images before and after radiation therapy at a ratio of 1:1. The patients presented at Yunnan Cancer Hospital between September 2019 and March 2023 and were complicated with radiation pneumonitis after radiation therapy. The CT images before radiation therapy were used as negative samples, while the CT images after radiation therapy were used as positive samples. Their ages ranged from 30 to 72 years, with the median age being 48 years, and the majority of patients underwent modified radical mastectomy, while a minority received breast-conserving surgery, with all of the patients having no smoking history. Their TNM clinical stages ranged from I to III, with Stage II and Stage III accounting for the majority. The molecular subtypes were predominantly Luminal A and Luminal B, and all patients received intensity-modulated radiation therapy (IMRT). Patients meeting the following criteria were included in the study: 1) patients who had pathologically confirmed breast cancer, 2) patients whose diagnosis of radiation pneumonitis followed the GBZ110–2002 guidelines, 3) patients with complete pre- and post-radiotherapy imaging and clinical data, and 4) patients without severe cardiac or pulmonary disease and other contraindications to radiotherapy. The dataset was randomly divided into a training set (n = 117) and a test set (n = 29) at a ratio of 8:2.

### 2.2 CT images and ROI acquisition

**Figure 1:**
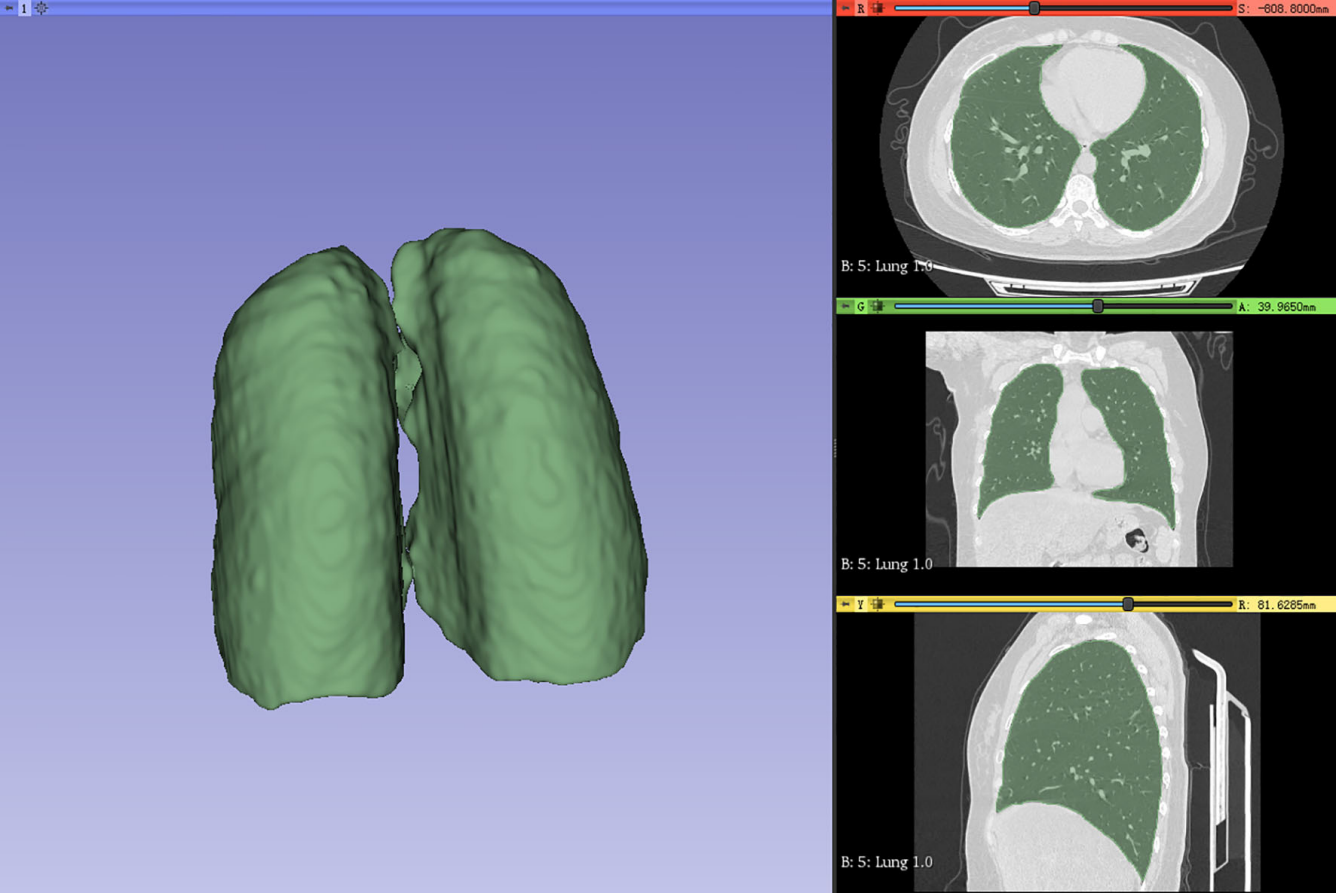
Whole lung (ROI) outlined by physicians. ROI, region of interest.

In this study, a Siemens (Somatom Force) third-generation dual-source CT system was utilized for image scanning of patients with breast cancer with a layer thickness of 1–2 mm, a tube voltage of 120 kV, a tube current of 30 mAs, an image size of 512 × 512 pixels, and image reconstruction algorithm(s) using Filtered Back Projection. The scanning range included complete and clear lung organs. The ROI was delineated by two clinical oncologists using the 3D slicer 4.11 software (15) with the whole lung delineated layer by layer, and the required ROI was reviewed and corrected by a senior radiologist (as shown in **Figure 1**).

### 2.3 Radiomic feature extraction

**Figure 2:**
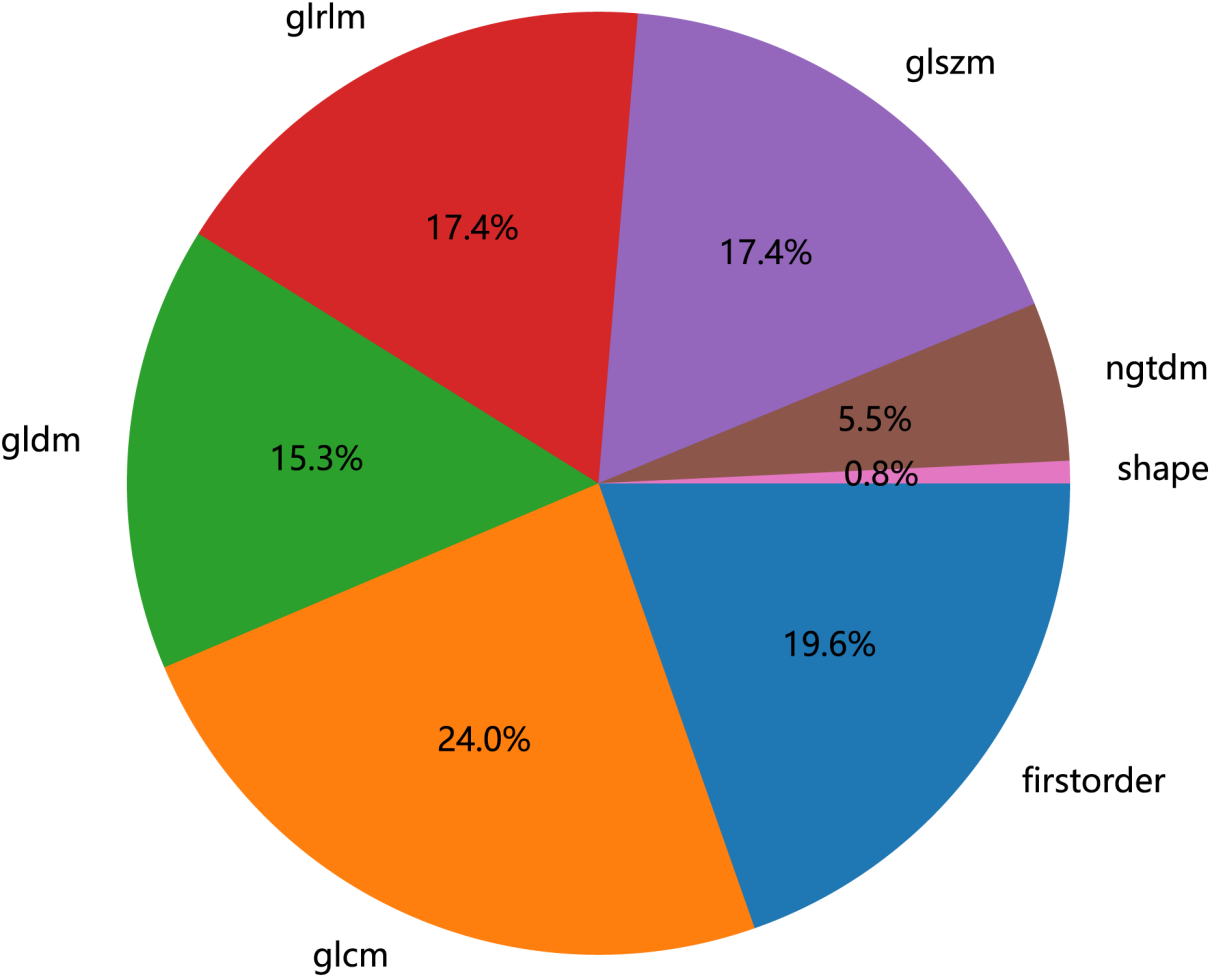
Number and ratio of handcrafted features, among which first-order stands for first-order features, shape for shape features, glcm for gray-level co-occurrence matrix features, glszm for gray-level size zone matrix features, glrlm for gray-level run length matrix features, ngtdm for neighboring gray tone difference matrix features, and gldm for gray-level dependence matrix features.

To eliminate the influence of image voxel size variation as much as possible, the images were resampled to 3 * 3 * 3 mm before extracting features in this study. The Radiomics package (http://pyradiomics.readthedocs.io) extracted radiomic features from the ROI (16). Nine image types were used for feature extraction: original, Laplacian of Gaussian (LoG), wavelet, LBP3D, exponential, square, square root, logarithm, and gradient. The sigma values of LoG were 1, 2, and 3. Wavelet included eight filters: wavelet-LLH, wavelet-LHL, wavelet-LHH, wavelet-HLL, wavelet-HLH, wavelet-HHL, wavelet-HHH, and wavelet-LLL. A total of 1,834 radiomic features were obtained, including 14 shape features (shape), 360 first-order features, 440 gray-level co-occurrence matrix (GLCM) features, 280 gray-level dependence matrix (GLDM) features, 320 gray-level run length matrix (GLRLM) features, 320 gray-level size zone matrix (GLSZM) features, and 100 neighboring gray tone difference matrix (NGTDM) features, as shown in **Figure 2**. The details of the features can be found in **Supplementary File S1**.

### 2.4 Feature selection

**Figure 3:**
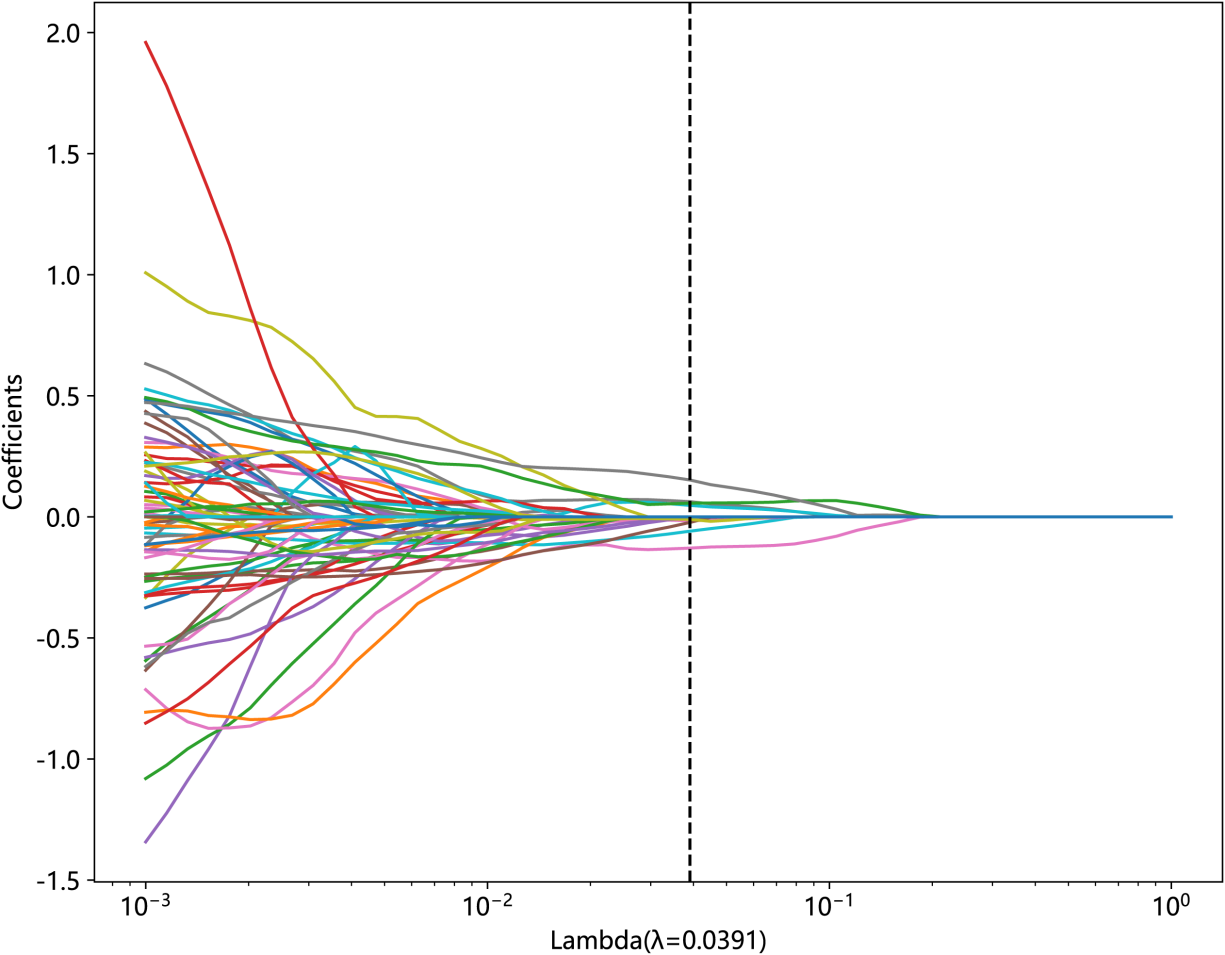
Relationship between lambda and regression coefficients (the optimal value of lambda is 0.0391).

**Figure 4:**
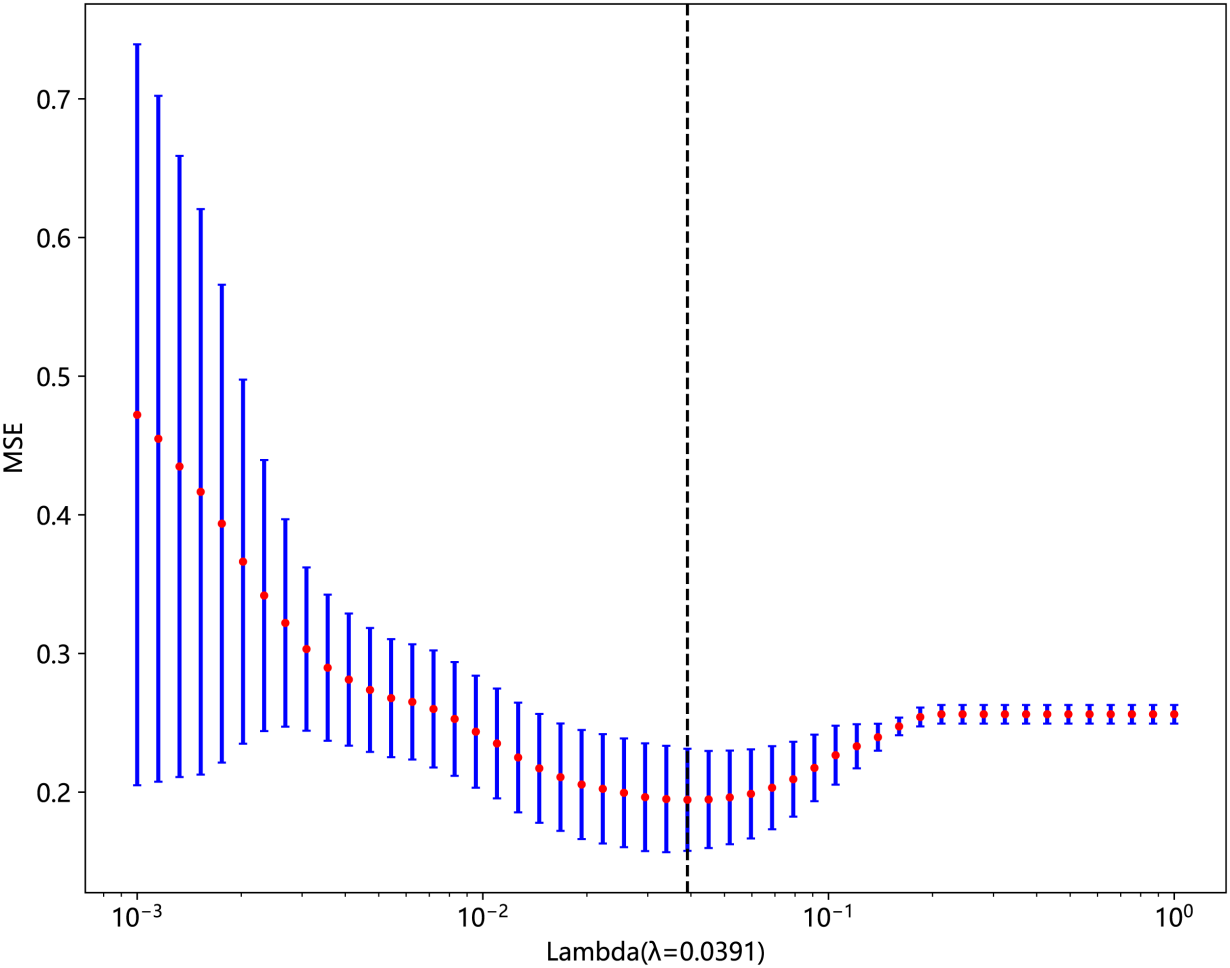
The MSE of LASSO regression.

We used Z-score to standardize the extracted features and performed data screening in three ways: statistical analysis, correlation analysis, and least absolute shrinkage and selection operator (LASSO) on the standardized data. First, we used the Mann–Whitney U-test statistical test for radiomic feature selection and retained 328 radiomic features with the p-value < 0.05. Second, we performed Spearman’s rank correlation analysis on the 328 radiomic features to eliminate redundant features with high repeatability, as shown in **Supplementary Material Figure 1**, and retained one of the two features with a correlation coefficient greater than 0.9, yielding a total of 82 features. Finally, we constructed the 82 obtained features using the LASSO model, which compressed the coefficients of the features by adjusting the weights λ and changed the coefficients of some of the features to zero to achieve feature selection. Our study performed a 10-fold cross-validation to find the optimal values, as shown in **Figures 3** and **4**, and we retained the features with non-zero coefficients for fitting the regression model and combined them into a radiomic signature. The optimal value of λ in this study was 0.0391.

### 2.5 Model construction and evaluation metrics

Our study utilized the sklearn package in Python 3 to build machine learning models. Specifically, we applied eight different algorithms: logistic regression (LR), support vector machine (SVM), K-nearest neighbors (KNN), random forest (RF), Extra Tree (ET), XGBoost, LightGBM, and multilayer perceptron (MLP). All of them were employed to discriminate RP, using four different metrics—accuracy, the area under the receiver operating characteristic curve (AUC), sensitivity, and specificity—and ROC curves to further assess their effectiveness.

## 3 Results

### 3.1 Radiomic feature filtering

**Figure 5:**
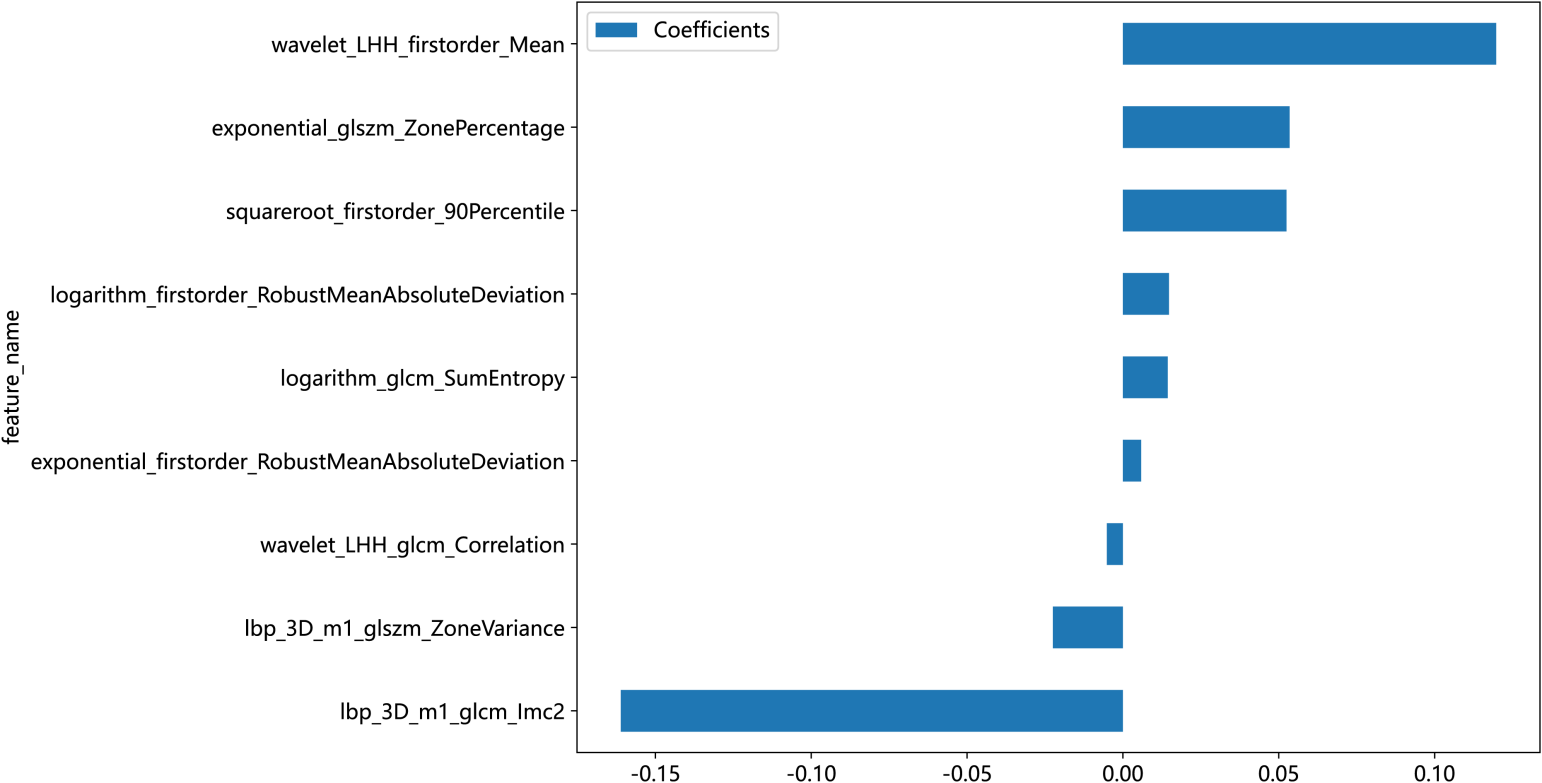
Weights of the nine radiomic features.

**Figure 6:**
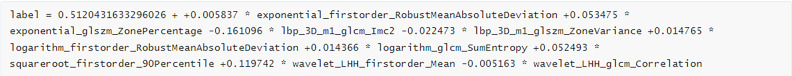
Graph of Rad-score results.

In this study, the 1,834 features extracted from the ROI were filtered through several steps, with nine retained radiomic features for the training and testing of the machine learning models, including four first-order features, two GLSZM features, and three GLCM features, as shown in **Table 1**. In order to show the weight of each feature more visually, the weight histogram and Rad score of the nine features were plotted, as shown in **Figures 5** and **6**.

**Table 1:** Radiomic features obtained after LASSO dimensionality reduction.

Feature type	Features	Feature weights
exponential_firstorder	RobustMeanAbsoluteDeviation	0.005837
exponential_glszm	ZonePercentage	0.053475
lbp_3D_m1_glcm	Imc2	−0.161096
lbp_3D_m1_glszm	ZoneVariance	−0.022473
logarithm_firstorder	RobustMeanAbsoluteDeviation	0.014765
logarithm_glcm	SumEntropy	0.014366
squareroot_firstorder	90Percentile	0.052493
wavelet_LHH_firstorder	Mean	0.119742
wavelet_LHH_glcm	Correlation	−0.005163

LASSO, least absolute shrinkage and selection operator.

### 3.2 Radiomics model prediction

**Figure 7:**
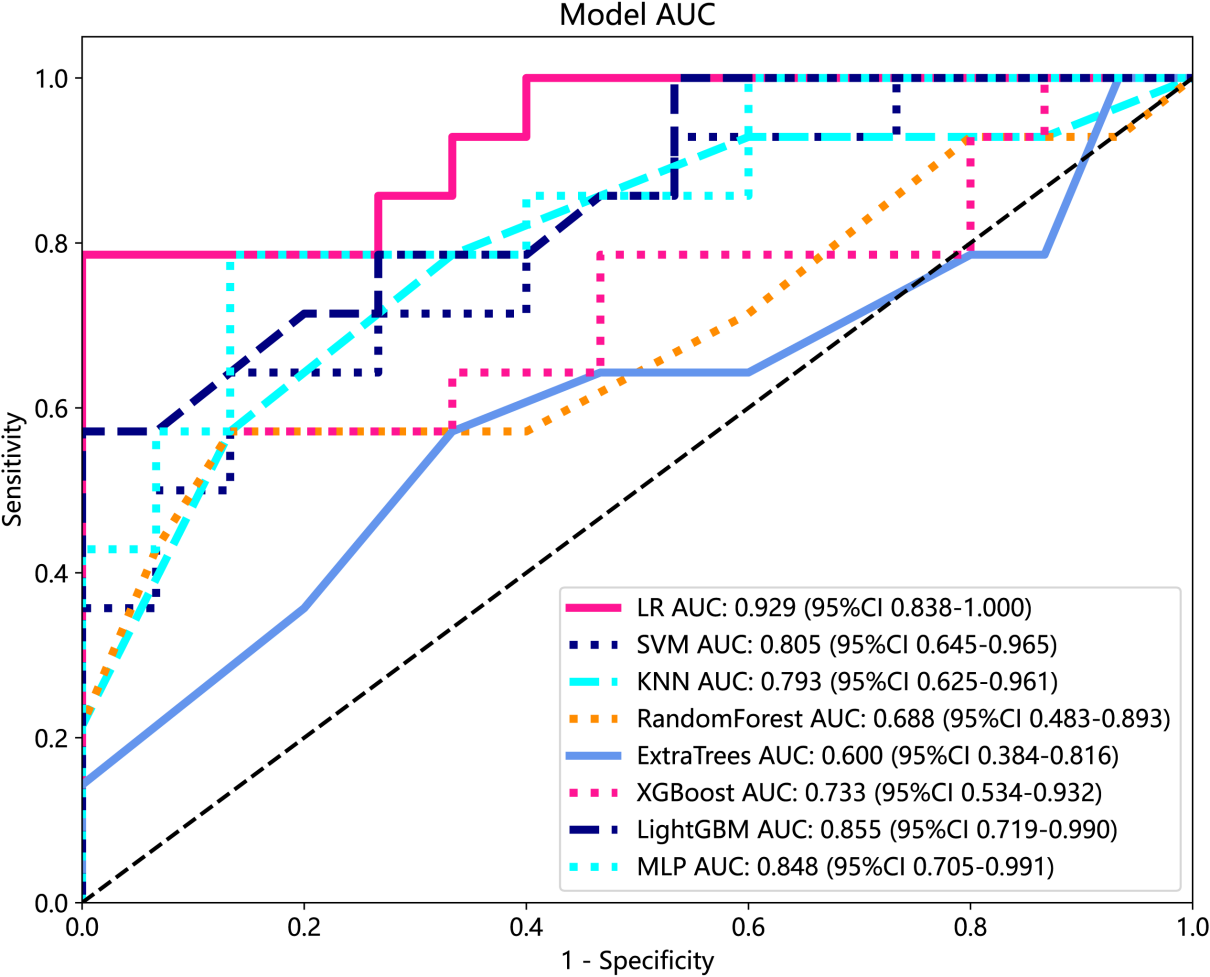
ROC analysis of different models on Rad signature. ROC, receiver operating characteristic.

We developed eight machine learning models to predict radiation pneumonitis based on selected radiomic features, as shown in **Table 2**. The SVM model demonstrated the highest accuracy on the test set, while the LR model achieved the best overall predictive performance, indicated by the highest AUC and specificity. Most of the models, except the ET model, showed robust diagnostic potential with AUC scores exceeding 0.7, confirming that radiomics-based machine learning can effectively identify radiation pneumonitis in breast cancer patients. ROC curves for these models further illustrate their comparative performances (**Figure 7**).

**Table 2:** Predictive performance of eight models in the training cohort and test cohort.

Model	Training cohort (n = 116)				Testing cohort (n = 29)			
	Accuracy	AUC (95% CI)	Sensitivity	Specificity	Accuracy	AUC (95% CI)	Sensitivity	Specificity
LR	0.761	0.83 (0.756–0.903)	0.542	0.983	0.897	0.929 (0.8383-1.000)	0.786	1
SVM	0.812	0.901 (0.849–0.952)	0.712	0.914	0.901	0.805 (0.645–0.965)	0.643	0.867
KNN	0.786	0.852 (0.787–0.916)	0.678	0.897	0.724	0.793 (0.625–0.961)	0.786	0.667
Random forest	0.983	0.999 (0.997–1.000)	0.966	1	0.724	0.688 (0.483–0.893)	0.571	0.867
Extra Tree	1	1 (1.000–1.000)	1	1	0.621	0.6 (0.384–0.816)	0.571	0.667
XGBoost	1	1 (1.000–1.000)	1	1	0.793	0.733 (0.534–0.932)	0.571	1
LightGBM	0.872	0.942 (0.904–0.980)	0.881	0.862	0.793	0.855 (0.719–0.990)	0.571	1
MLP	0.778	0.818 (0.741–0.894)	0.797	0.759	0.828	0.848 (0.705–0.991)	0.786	0.867

SVM, support vector machine; KNN, K-nearest neighbor; MLP, multilayer perceptron; AUC, area under the receiver operating characteristic curve.

## 4 Discussions

Previous studies on RP have mainly focused on its prediction and diagnosis using dosimetric parameters, pulmonary metabolic activity, etc. (17, 18). However, this predictive approach’s precision and robustness still need to be improved. With the development of artificial intelligence, radiomics provides a novel approach for diagnosis and prognosis in clinical practice.

Numerous studies have confirmed that radiomics-based models exhibit robust and superior performance across multiple cancer-related prediction and classification tasks (19, 20). Therefore, radiomics can non-invasively extract imaging information and analyze its potential clinical value in a high-throughput manner to establish a practical model for prognosis and discrimination in clinical applications, thus potentially enabling the development of non-invasive differential models for the diagnosis of RP (11, 21).

Current studies have demonstrated that the integration of radiomic features with machine learning algorithms substantially improves predictive accuracy for RP differentiation in radiotherapy settings, thereby establishing a foundation for developing clinically applicable RP diagnostic models (21, 22). However, most of these radiomics studies focused on lung cancer, where the tumors’ anatomical location and adjacent structures may present radiomic feature alterations in the lung, affecting the prediction results and the validity of the extracted features for radiolucency after radiotherapy for lung cancer. RP after the radiotherapy for breast cancer is mainly in the first to second intercostal space, and the lung is less affected by the tumor. Consequently, the generalizability and robustness of the extracted features based on lung cancer may be affected to different degrees. Therefore, there is a need to personalize the diagnosis and prognosis of RP for breast cancer patients.

Our study aimed to detect radiomics changes on CT images before and after radiotherapy and explore the feasibility of employing them to discriminate the occurrence of RP in patients with breast cancer after radiotherapy. We utilized various machine learning models to diagnose the occurrence of RP after radiotherapy. It is noteworthy that we initially personalize the diagnosis of RP by comparing radiomics changes on CT images before and after radiation therapy in breast cancer patients who develop RP.

Currently, the investigators’ selection of deep learning models or classification algorithms in their study is usually determined by their experience, or they consider factors such as the frequency of being cited in the literature, data characteristics and quality, and the availability of simple implementation (23). Parmar et al. (24) found that the choice of different classification methods had a significant impact on the model performance (34.21% of the total variance of the models). Therefore, the appropriate choice of machine learning model is crucial for the prediction. In this study, eight machine learning models were constructed for prediction, and the results showed that, except for the Extra Tree model, the seven other machine learning models presented AUC values higher than 0.7 in the test cohort. The LR model was optimal for AUC, sensitivity, and specificity metrics in the test set, with an AUC value of 0.929, which indicated that the radiomic features obtained from CT images could effectively individualize the diagnosis of RP in breast cancer patients. It can assist clinical oncologists in rapid diagnosis and the adjustment of treatment plans. A total of nine radiomic features were selected in this study, including five textural features, which may be explained by the fact that lung texture is altered to varying degrees in the presence of RP, which is consistent with the assumption that textural features are more suitable for detecting tissue structural heterogeneity on imaging (25). Mean, 90Percentile, and RobustMeanAbsoluteDeviation were selected as the first-order features, probably because of the significant grayscale changes in the lungs in the presence of RP, which may lead to changes in the mean grayscale values and 90Percentile and RobustMeanAbsoluteDeviation. Cunliffe et al. (26) explored the correlation of radiomic features with radiotherapy dose and the occurrence of RP based on CT images of patients undergoing radiotherapy for lung cancer. Similarly, they found that there was a statistically significant relationship between MEAN features in first-order features and the occurrence of RP (p < 0.0025), which is similar to the results of the present study. It is worth noting that most of the radiomic features extracted in this study were features obtained by the wavelet extractor and local binary pattern (LBP) extractor, and the wavelet transform can extract features from the frequency domain and effectively enhance the texture features of CT. Also, LBP is an operator used to describe local texture features of images. Jiang et al. (27) used a CT-based wavelet transform radiomics method to grade lung lesions caused by COVID-19, and the results showed that wavelet transform could enhance CT texture features, and wavelet transform radiomics based on CT images can be used to assess the grading of COVID-19-induced lung lesions effectively. Additionally, similar findings have proved that all radiomic features with wavelet filters that were selected using LASSO regression were important predictors for the prediction of RP grade (21). Therefore, combined with the results of this study, it can be inferred that texture features are an important component for the diagnosis of RP in breast cancer patients and that feature extractors such as LBP and wavelet that can enhance or amplify texture features may be more advantageous, and it is recommended that extractors that can focus on texture features should be added when performing radiomic feature extraction for RP in breast cancer.

There is still some room for improvement in this study. This study only used radiomic features for diagnosing RP. In future studies, it is necessary to consider the influence of patients’ clinical traits, such as age, gender, smoking status, and lung disease (28). Dose parameters during treatment planning can affect the incidence rate of RP and should also be used as a reference (29–32).

Furthermore, future research should implement clinical stage stratification to identify stage-specific radiomic signature alterations. Other approaches, such as visual assessment systems (e.g., radiological scoring scales), should be incorporated to augment the feature pool. Also, this study is a single-center study with limited samples, and the models’ robustness and generalization will be somewhat challenged; additional datasets and multi-center studies should be conducted in the future.

## 5 Conclusions

When RP occurs, radiomics is more likely to exhibit changes in texture features and first-order characteristics, and feature extractors that can focus on or amplify texture features, such as wavelets and LBP, may be more advantageous in discriminating the RP occurrence. Meanwhile, machine learning models based on radiomic features can effectively predict the RP occurrence.

## Data Availability

The original contributions presented in the study are included in the article/**Supplementary Material**. Further inquiries can be directed to the corresponding authors.

## Supplementary material

Supplementary File S1 Radiomics features.

Supplementary Figure 1 Cluster analysis of radiomics features (Darker colors represent more positive correlations between features, and lighter colors represent more negative correlations).
