# Correction: Personalized diagnosis of radiation pneumonitis in breast cancer patients based on radiomics

**DOI:** 10.3389/fonc.2025.1674168

**Published:** 2025-08-26

**Authors:** Xiaobo Wen, Yutao Zhao, Wen Dong, Congbo Yang, Jinzhi Li, Li Sun, Yutao Xiu, Chang’e Gao, Ming Zhang

**Affiliations:** ^1^ School of Pharmacy, Qingdao University, Qingdao, China; ^2^ Department of Radiotherapy, Yunnan Cancer Hospital, the Third Affiliated Hospital of Kunming Medical University, Kunming, Yunnan, China; ^3^ Cancer Institute of The Affiliated Hospital of Qingdao University and Qingdao Cancer Institute, Qingdao, China; ^4^ Department of Medical Oncology, The First Affiliated Hospital of Kunming Medical University, Kunming, China

**Keywords:** breast cancer, radiomics, radiation pneumonitis, machine learning, artificial intelligence

In the published article, there was an error in the legend for **Figure 4** as published. The original legend did not match the actual content of the figure. The corrected legend appears below.

**Figure 4:**
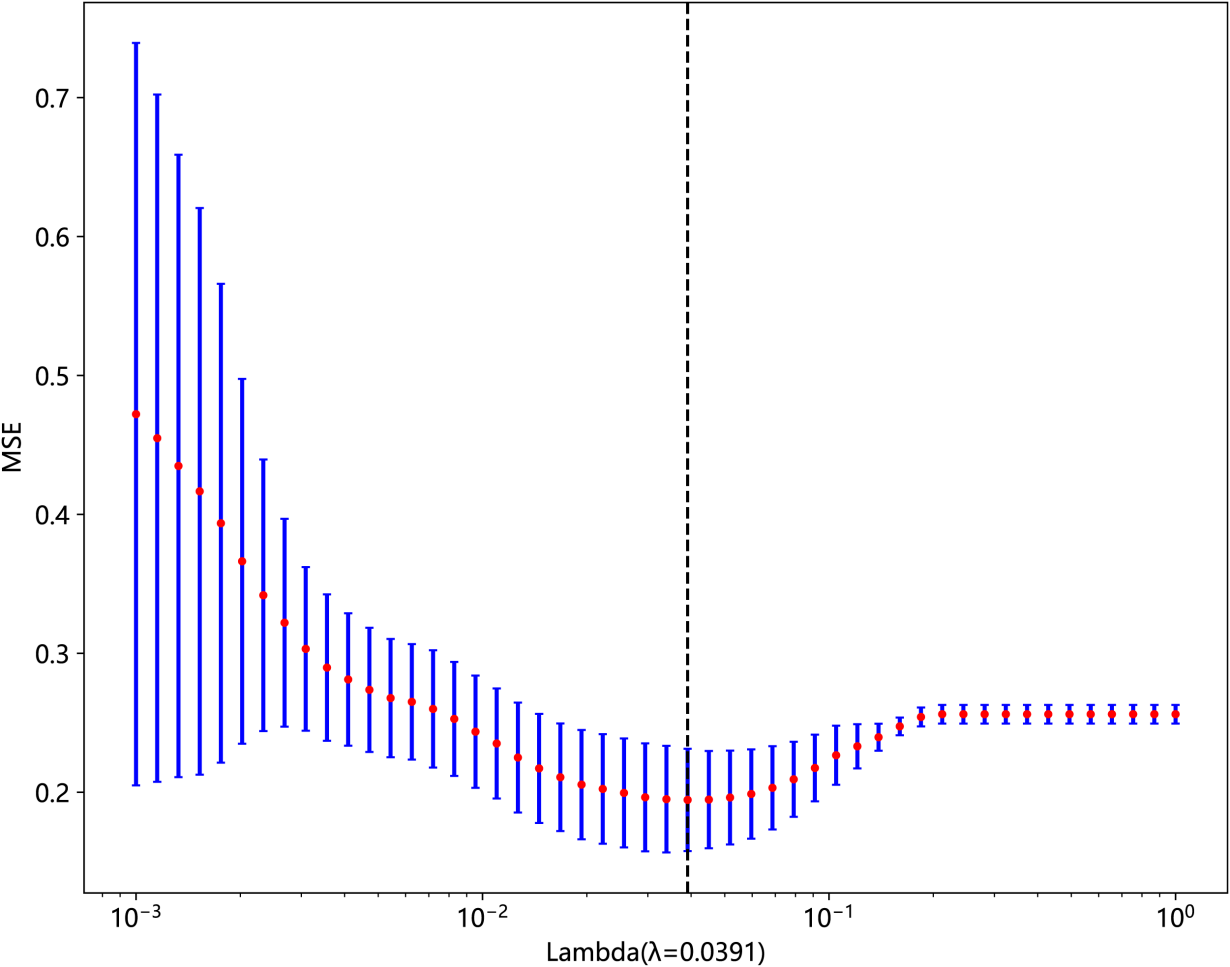
The MSE of LASSO regression.

“The MSE of LASSO regression.”

The original version of this article has been updated.

